# The Immunomodulatory Activity of *Staphylococcus aureus* Products Derived from Biofilm and Planktonic Cultures

**DOI:** 10.1007/s00005-013-0240-3

**Published:** 2013-08-08

**Authors:** Beata Sadowska, Marzena Więckowska-Szakiel, Małgorzata Paszkiewicz, Barbara Różalska

**Affiliations:** Department of Immunology and Infectious Biology, Institute of Microbiology, Biotechnology and Immunology, Faculty of Biology and Environmental Protection, University of Lodz, Banacha 12/16, 90-237 Lodz, Poland

**Keywords:** Staphylococci, Biofilm, Modulins, Phagocytes, Cytokines

## Abstract

Biofilms are probably one of the most common structures formed by microorganisms in various environments. The higher resistance of such microbial communities to stress conditions, including antibiotics and host immune response, is recently extensively studied. However, the weak activity of phagocytic cells against microbial biofilm is not yet fully understood and explained. The aim of this study was: (1) a qualitative and quantitative comparison of cell components/products released from *Staphylococcus aureus* biofilm or planktonic cultures, (2) evaluation of the influence of such cell components/products on murine leukocytes secretory function. For this, mouse peritoneal leukocytes were stimulated with biofilm or planktonic staphylococcal cultures or their acellular filtrates, and then the production of cytokines (TNF-α, IL-6, IL-10, MCP-1 and MIP-1α), hemolytic activity and staphylokinase (SAK) production was determined. It was found that similar staphylococcal components/products possessing the immunomodulatory properties, were present in both, biofilm and planktonic filtrates. Moreover, these compounds were similarly active in the stimulation of TNF-α and MCP-1 release from leukocytes. The hemolytic activity and SAK release by planktonic and biofilm cultures were also comparable. What is interesting, stronger stimulatory activity of biofilm-derived components/products of clinical *S. aureus* strains in the case of MIP-1α, IL-6 and IL-10 was noticed. On the other hand, taking into consideration the reference strains, MIP-1α production was enhanced by “planktonic filtrates”. Thus, in our study it was proved, first of all, that biofilm is not a structure fully separated from the external environment. Second, the influence of these *S. aureus* constituents/metabolites on leukocytes seems to be more strain-dependent than culture phenotype-dependent. The lack of one common profile of biofilm and planktonic *S. aureus* cultures/filtrates biological activity indicates that the disturbances in cytokines’ production could not be the only reason for the so-called “frustrated phagocytosis”, connected with enhanced biofilm resistance.

## Introduction

Biofilms—the sessile cell communities, encased in an extracellular polymeric substance (EPS), are probably one of the most common structures formed by microorganisms in various environmental conditions, including animal and human bodies. The participation of microbial biofilms in the pathogenesis of many disease processes is well documented. Initially, it was thought that biofilms were associated mostly with invasive medical procedures using biomaterial devices. However, recent research has shown the occurrence of biofilms in many other conditions, including chronic wound infections (e.g. postoperative, bedsore, burn-related, diabetic), chronic otolaryngological, recurrent genitourinary or bronchopulmonary infections, even systemic infections (Bjarnsholt et al. 2008; Leid 2009; Vlassova et al. 2011). The factors which contribute to higher resistance of bacterial biofilm to environmental stress conditions, including antibiotics and host immune response, are an essential subject of modern experimental research. Nevertheless, the phenomenon of weak activity of phagocytic cells against microbial biofilm (called “frustrated phagocytosis”) is not yet fully understood and explained (Leid 2009; Thurlow et al. 2011; Vlassova et al. 2011). For many years it was believed that phagocytes do not penetrate biofilm through EPS. However, this opinion has begun to be negatively verified during numerous studies based on microscopic observations, demonstrating attachment and penetration of matured bacterial biofilm by human leukocytes (Leid et al. 2002; Leid 2009). Recent in vivo research on a mouse model indicates an evasion of innate immunity by staphylococcal biofilm. Significant reduction in the pro-inflammatory cytokines level and limited leukocyte/phagocyte infiltration into biofilm were observed during catheter-associated infections (Thurlow et al. 2011). This suggests a weaker induction of host inflammatory response by sessile bacteria compared to their planktonic form. A slower metabolic activity of bacterial cells in biofilm, especially those located in its deeper layers, may be one of the reasons. Also, the biofilm structure should be taken into consideration due to the EPS, which can block the access of host receptors belonging to the group of pattern recognition receptors recognized pathogen associated molecular patterns.

It was the reason why we wanted to check on in vitro model the secretion of cytokines/chemokines by freshly isolated murine peritoneal leukocytes, in response to both staphylococcal cultures (sessile/planktonic) and acellular filtrates obtained from them. We address a question concerning *Staphylococcus aureus* biofilm as a source of bacterial components/products, which may modulate and attenuate the host immune response. Qualitative and quantitative composition of the filtrates obtained from staphylococcal biofilm and planktonic cultures was evaluated. Considering the crucial role of cytokines/chemokines in the development of innate and adaptive immunity, the production of selected pro-inflammatory cytokines or products with suppressory activity, was tested.

## Materials and Methods

### Bacterial Cultures and Their Filtrates

Two reference *S. aureus* strains: 8325-4 (high α-hemolysin (Hla) expression; SAK^−^), Wood 46 (Hla overexpression, SAK^+^) and two clinical strains isolated from the lungs of cystic fibrosis patients: Sa-α11 (SAK^−^), Sa-α21 (SAK^+^) were grown for 24 h at 37 °C in tryptic soy broth (TSB; BTL, Poland). Then, bacterial cultures were diluted 1:50 in TSB and incubated for the next 24 h in the tubes with aeration as a planktonic culture or on 24-well plates in stable conditions to form biofilm. To obtain acellular filtrates, planktonic or biofilm *S. aureus* cultures were incubated for the next 4 h, as described before, preceded by the exchange of the medium into a fresh one. Finally, bacterial cultures were centrifuged (3,000 rpm, 10 min), the supernatants were collected, filtered (0.22 μm; Millipore, Germany) and stored at −20 °C until testing.

### Staphylococcal Density in Planktonic and Biofilm Cultures

To count the number of bacterial cells in their planktonic or biofilm form, staphylococcal cultures were prepared as described above. After final centrifugation and removal of supernatants, bacteria in planktonic form in the tubes were resuspended in 1 ml of fresh TSB. Similarly, 1 ml of fresh TSB was added to the biofilm of staphylococci formed in the wells of a plate, and then bacterial cells were dislodged mechanically by scraping the bottom and vigorous aspiration and release of the medium. Obtained bacterial suspensions were diluted from 10^−1^ to 10^−10^ in phosphate buffered saline (PBS; Biomed, Poland) preceded by intensive vortexing. Then 100 μl of staphylococcal suspensions (10^−7^–10^−10^) was cultured on agar plates and colony forming units (CFU) were counted after 24 h incubation at 37 °C. The experiment was performed twice and each bacterial culture was prepared in duplicate. The density of initial staphylococcal suspensions was calculated using the average value of CFU.

### Analysis of Staphylococcal Cells Components/Products

The presence of peptidoglycan (PG), lipoteichoic acids (LTA), Hla and staphylokinase (SAK) in bacterial cultures’ filtrates was detected. To determine the content of PG, the silk larva plasma test (Wako, Japan) was used according to the manufacturer’s procedures. An individually designed protocol of the enzyme-linked immunosorbent assay (ELISA) was prepared to estimate the presence of LTA. Mouse monoclonal antibody against LTA (Hycult Biotechnology, The Netherlands) diluted 1:50 in PBS with 0.5 % bovine serum albumin and goat polyclonal antibody against mouse IgG and IgM horseradish peroxidase (HRP)-conjugated (Hycult Biotechnology, The Netherlands) diluted 1:1,000 in the same buffer were used. The activity of secreted staphylococcal Hla was measured spectrophotometrically (*A*_550_), using 5 % suspension of sheep erythrocytes. It was calculated as values relative to the absorbance of control cells after saponin treatment (at 1 % final concentration), regarded as 100 % of hemolysis. The presence of Hla in the filtrates was confirmed by Western blot, using rabbit monoclonal antibody against Hla (Sigma, Germany) diluted 1:5000 in PBS and swine polyclonal antibody against rabbit IgG HRP-conjugated (Dako, Denmark) diluted 1:1,000 in the same buffer. SAK activity was determined by measuring specific plasmin substrate hydrolysis in the presence of 1 μM plasminogen in Tris/HCl buffer (0.14 M NaCl, 1.5 M Tris–HCl, pH 7.2), as described earlier (Więckowska-Szakiel et al. 2007).

### Mouse Leukocytes

Balb/c mice aged 3–6 months, bred at The Animal House of the Institute of Microbiology, Biotechnology and Immunology, University of Lodz were intraperitoneally injected with 1 ml of 10 % protease-peptone. After 18 h or 72 h the mice were sacrificed by cervical dislocation under ether anaesthesia and peritoneal exudates with the prevalence of, respectively, polymorphonuclear (PMN) cells or macrophages (Mo) were obtained. Mouse peritoneal cavity was washed with PBS containing 2 % of calf serum (Biomed, Poland) and 1 % of ethylenediamine tetraacetic acid (Sigma, Germany). All experiments were performed according to the ethical guidelines of the Local Committee on the Ethics of Animal Research (resolution No. 25/ŁB 518/2010). The obtained cells were washed and resuspended in RPMI-1640 medium (Sigma, Germany) with 5 % of fetal calf serum (FCS; Cytogen, Poland). Each time, the density and the viability of the cells were estimated using the trypan blue (Merck, Germany) exclusion assay. The composition of the obtained peritoneal cell suspensions was determined by flow cytometry (FACS counter; Becton–Dickinson, USA). The cells (3 × 10^6^ cells/ml, 100 μl) were marked with the antibodies specific to selected surface clusters: rat anti-mouse CD11b: RPE labelled, rat anti-mouse CD14: RPE labelled and rat anti-mouse F4/80 antigen: FITC labelled (Serotec, UK) (5 μl of antibody, 30 min on ice). FlowJo software was used for the analysis of flow cytometry data.

### Leukocytes Supernatants

The suspensions of leukocytes (3 × 10^6^ cells/ml) were prepared in the twofold concentrated RPMI-1640 supplemented with 10 % FCS, l-glutamine (4 mM), NaHCO_3_ (4 mg/ml), penicillin (200 U/ml), streptomycin (200 μg/ml; all components were purchased from Sigma, Germany) and polymyxin B (100 μg/ml; Serva, UK). Then, the leukocytes were mixed with each bacterial filtrate (ratio 1:1) or added to each bacterial culture (biofilm or planktonic) in the same ratio and incubated at 37 °C with 5 % CO_2_. The cells were stimulated by filtrate components for 2 and 24 h. Direct leukocyte stimulation by live *S. aureus* cultures lasted 4 h, which was the same time period as that required to obtain the filtrates from bacterial cultures. Finally, the leukocytes were centrifuged (2,400 rpm, 10 min), the culture supernatants were collected and stored at −20 °C until analyses.

### Assessment of the Cytokines Concentration

The presence of TNF-α, IL-6, IL-10, JE/MCP-1 and MIP-1α in the supernatants of leukocytes treated with *S. aureus* cultures (biofilm or planktonic) or their filtrates was investigated. The ELISA Development Kits specific for murine TNF-α, JE/MCP-1, MIP-1α (PeproTech, France) and murine IL-6 or IL-10 Eli-pair (Diaclone, France) were used according to the manufacturer’s instruction. The data are presented as the means ± SD from minimum two independent experiments with duplicates each.

### Bacterial Proteases Activity Against Cytokines

In our previous study we evaluated the proteolytic activity of the four tested staphylococcal strains using agar plates containing casein (Difco, USA). *S. aureus* 8325-4, Wood 46 and α11 exhibited strong proteolytic activity. In the case of *S. aureus* α21 proteolytic activity was observed only in the presence of the antibiotics (data not shown). Therefore, it could have been suspected that proteases are secreted into staphylococcal filtrates and may act against cytokines. To check such a possibility in the simplest way, IL-10 (250 pg/ml) was mixed (ratio 1:1) with each tested bacterial filtrate or with fresh TSB medium (control) and incubated for 2 and 24 h at 37 °C with 5 % CO_2_. Then, IL-10 concentration in the prepared samples was estimated using murine IL-10 Eli-pair ELISA test, according to the manufacturer’s instruction.

### Statistics

Mann–Whitney *U* test was used to evaluate the statistical significance of the results of the cytokine/chemokine production by induced phagocytes. The differences with *p* < 0.05 were considered as significant.

## Results

### Bacterial Cultures and Their Density

To standardize bacterial growth conditions, we made an evaluation of staphylococcal cell density using CFU method preceded by the culture which was prepared according to the procedure provided for these studies. The number of bacterial cells in the cultures of three *S. aureus* strains (8325-4, α11, α21) was similar and showed a mean of 4.5 × 10^8^ ± 1.9 × 10^8^ CFU/ml for planktonic cultures and 7.9 × 10^8^ ± 6.2 × 10^8^ CFU/ml for biofilm cultures. Only the density of *S. aureus* Wood 46 cultures was slightly higher, reaching a value of 2.4 × 10^9^ CFU/ml for planktonic culture and 3.7 × 10^9^ CFU/ml for biofilm culture. However, it did not increase the biological activity of their filtrates in comparison to the filtrates from the other tested strains with lower density of the bacterial cells in their starting culture. Most importantly, the number of bacterial cells within the planktonic and biofilm population of the same staphylococcal strain was comparable and reached, respectively, in planktonic and biofilm cultures: 4.3 × 10^8^ and 5 × 10^8^ CFU/ml for *S. aureus* 8325,4; 2.4 × 10^9^ and 3.7 × 10^9^ CFU/ml for *S. aureus* Wood 46; 7 × 10^8^ and 1.5 × 10^9^ CFU/ml for *S. aureus* α11; 3.1 × 10^8^ and 3.6 × 10^8^ CFU/ml for *S. aureus* α21, which enabled us to make a direct comparison of the biological activity of these two types of filtrates.

### Staphylococcal Components/Products Released to the Culture Medium

**Fig. 1:**
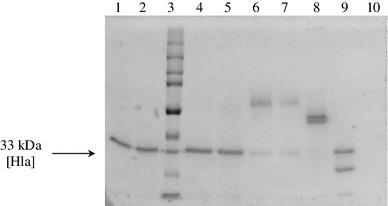
The presence of Hla in the filtrates obtained from planktonic (PL) or biofilm (B) *S. aureus* cultures. *1* Sa-8325-4 PL, *2* Sa-8325-4 B, *3* molecular mass standard, *4* Sa-Wood 46 PL, *5* Sa-Wood 46 B, *6* Sa-α11 PL, *7* Sa-α11 B, *8* Sa-α21 B, *9* Hla standard, 20 μg/ml (Sigma, Germany), *10* negative control (TSB medium)

The release of PG from *S. aureus* 8325-4 and Sa-α11 reached the highest concentrations, respectively, in biofilm/planktonic cultures, 178.1/132.1 ng/ml and 128.8/157.0 ng/ml. It was shown that the clinical strains released 2.1–9.5 times more LTA during planktonic growth and 10.9–20.0 times more while growing as biofilm in comparison to the reference strains (Table 1). The filtrates from *S. aureus* 8325-4 and Wood 46 cultures (both: biofilm and planktonic) expressed stronger hemolytic activity than these obtained from Sa-α11 and Sa-α21 cultures (Table 1). The presence of Hla in the above filtrates was confirmed by Western blot (Fig. 1). Since *S. aureus* 8325-4 and Sa-α11 were considered as SAK negative, only SAK levels produced by two other strains were compared. They reached, respectively, for biofilm/planktonic cultures of *S. aureus* Wood 46 and Sa-α21, 0.7 ± 0.02/2.6 ± 0.75 μg/ml and 0.6 ± 1.0/2.8 ± 0.51 μg/ml and were assessed as high production of SAK in planktonic cultures and its low production in biofilm cultures. It means that SAK production was repressed in biofilm formed by SAK positive staphylococci.

**Table 1:** The concentrations of LTA and the activity of Hla secreted to the media during planktonic (PL) and biofilm (B) growth of staphylococci

Type of growth	LTA (ng/ml) ± SD		Hemolytic activity (%) ± SD	
	PL	B	PL	B
*S. aureus* 8325-4	42.8 ± 13.4	10.9 ± 5.7	74.9 ± 7.0	90.0 ± 4.1
*S. aureus* Wood 46	64.4 ± 46.0	19.9 ± 19.9	65.7 ± 5.3	100.0 ± 37.1
Sa-α11	237.4 ± 44.5	219.2 ± 120.5	39.9 ± 4.0	78.4 ± 23.3
Sa-α21	355.1 ± 202.3	216.5 ± 119.7	61.7 ± 9.9	58.0 ± 17.1

All data are representative (mean value) of those from two independent experiments

*SD* standard deviation

### Production of the Chemokines (MIP-1α, JE/MCP-1)

**Fig. 2:**
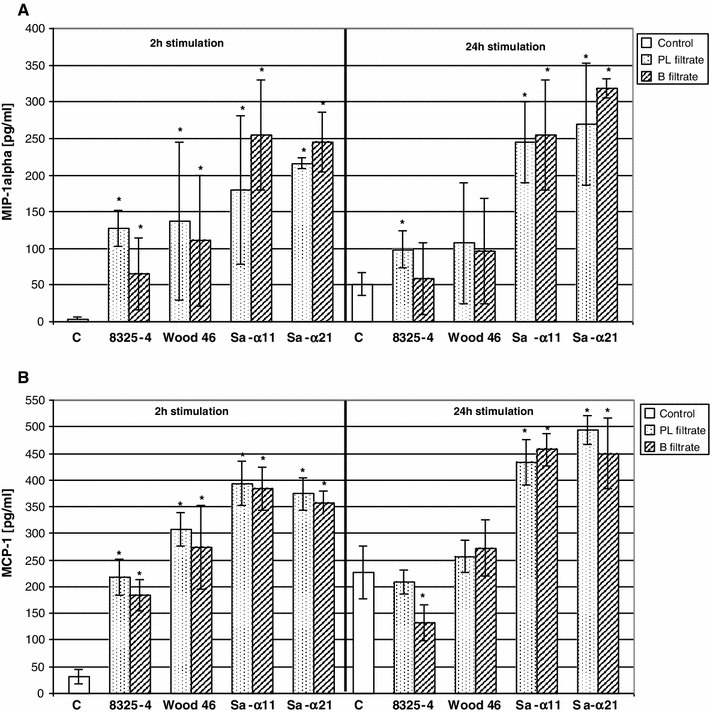
MIP-1α (**a**) and MCP-1 (**b**) production by mouse PMN after 2 and 24 h stimulation with bacterial filtrates obtained from planktonic (PL) or biofilm (B) cultures of *S. aureus* strains (8325-4, Wood 46, Sa-α11, Sa-α21. **c** Unstimulated control cells. All data are the means ± SD. *A significant differences (*p* < 0.02) in chemokines’ concentration compared to control

The percentage of the leukocyte type in peritoneal exudates was calculated for, respectively, PMN and Mo, as 79.9 and 94.9 %. Strong stimulatory effect of bacterial cultures’ filtrates (biofilm or planktonic) on MIP-1α and JE/MCP-1 production after 2 and 24 h of these cells’ induction was observed. Both types of the cells (PMN and Mo) responded similarly, so we decided to show the results only for neutrophils (Fig. 2). A statistically significant increase in MIP-1α secretion was observed in almost all tested layouts after both times of cell induction (2/24 h), in comparison to control (unstimulated) cells. Higher concentrations of MIP-1α were noticed for the leukocytes stimulated by the filtrates of clinical *S. aureus* strain (Sa-α11, Sa-α21) cultures than those derived from reference strains (Fig. 2a). The same observations were made for MCP-1 production (Fig. 2b).

The secretion of these chemokines after the contact of the leukocytes with live *S. aureus* strains growing as biofilm or planktonic cultures was also examined. The period of this contact was exactly the same as that required to obtain bacterial cultures’ filtrates (4 h). As it was shown in Table 2, the concentrations of MIP-1α and MCP-1 were even higher than those obtained after cell stimulation with bacterial filtrates. However, the profile of the leukocytes response was similar to the one observed previously: for both cell types the production of cytokines was stronger in reaction to clinical *S. aureus* strains, and there were no significant differences between biofilm and planktonic bacterial cultures’ forms in their stimulatory activity.

**Table 2:** The chemokines’ secretion by mouse PMN and Mo after their 4-h contact with live staphylococci growing as planktonic or biofilm form

Type of culture	MIP-1α (pg/ml) ± SD		MCP-1 (pg/ml) ± SD	
	PMN	Mo	PMN	Mo
*S. aureus* 8325-4				
PL	61.1 ± 3.7*	37.1 ± 1.7	491.8 ± 14.0*	391.8 ± 11.1*
B	43.9 ± 3.6*	37.2 ± 4.1	283.2 ± 23.6*	241.0 ± 13.1*
*S. aureus* Wood 46				
PL	62.8 ± 8.2*	49.8 ± 12.9*	396.3 ± 44.1*	393.4 ± 56.5*
B	87.4 ± 8.3*	67.1 ± 6.5*	365.9 ± 39.7*	367.3 ± 13.2*
Sa-α11				
PL	155.7 ± 10.4*	144.1 ± 17.0*	795.4 ± 55.9*	726.5 ± 24.8*
B	225.3 ± 10.9*	212.8 ± 9.0*	921.7 ± 14.5*	862.0 ± 15.1*
Sa-α21				
PL	389.6 ± 13.8*	352.1 ± 5.8*	1168.9 ± 10.3*	1106.9 ± 23.5*
B	354.9 ± 15.4*	292.5 ± 21.0*	1149.8 ± 10.6*	1092.2 ± 25.5*
Unstimulated cells (control)	6.4 ± 6.6	23.9 ± 10.6	37.6 ± 11.5	26.2 ± 10.0

All data are the means ± SD for four measurements

* A significant differences (*p* < 0.02) in chemokines’ concentration compared to control

### TNF-α, IL-6 and IL-10 Secretion

The secretion of TNF-α, IL-6 and IL-10 by PMN and Mo induced with acellular staphylococcal filtrates or live *S. aureus* strains growing as biofilm/planktonic forms was assessed. The obtained results are presented, respectively, in Tables 3 and 4. Both types of bacterial culture filtrates (biofilm/planktonic) showed similar stimulatory effect on TNF-α production by the peritoneal leukocytes. As was observed earlier, the filtrates of clinical strain (Sa-α11, Sa-α21) cultures were more potent stimulants (Table 3).

**Table 3:** The cytokines’ production by mouse PMN and Mo after stimulation with bacterial filtrates obtained from planktonic (PL) or biofilm (B) cultures of staphylococci

Type of supernatant	PMN		Mo	
	2 h	24 h	2 h	24 h
TNF (pg/ml) ± SD				
*S. aureus* 8325-4				
PL	221.7 ± 195.4	200.3 ± 93.2	169.9 ± 52.9	193.7 ± 18.9*
B	150.3 ± 125.8	155.6 ± 110.9	200.0 ± 25.2*	189.3 ± 20.6*
*S. aureus* Wood 46				
PL	161.5 ± 114.4	181.1 ± 100.9	197.7 ± 36.6*	174.3 ± 22.3*
B	139.3 ± 94.8	190.9 ± 110.1	219.6 ± 21.6*	183.2 ± 13.1*
Sa-α11				
PL	163.9 ± 63.6	507.9 ± 239.6*	180.5 ± 30.2*	192.0 ± 21.1*
B	180.3 ± 53.8	468.9 ± 93.1*	193.3 ± 20.8*	410.9 ± 109.3*
Sa-α21				
PL	236.3 ± 23.7*	618.1 ± 100.0*	204.7 ± 22.2*	304.0 ± 62.4*
B	206.0 ± 41.3*	621.4 ± 66.4*	202.4 ± 11.0*	392.5 ± 19.1*
Unstimulated cells (control)	152.1 ± 23.1	204.0 ± 70.5	116.9 ± 6.1	119.0 ± 12.5
IL-6 (pg/ml) ± SD				
Sa-α11				
PL	3.2 ± 6.2	120.1 ± 126.8	10.7 ± 6.6	49.0 ± 19.7*
B	28.3 ± 10.3*	646.4 ± 41.9*	128.1 ± 9.4*	515.7 ± 303.0*
Sa-α21				
PL	17.3 ± 14.9*	457.6 ± 110.1*	10.2 ± 11.9	449.3 ± 116.7*
B	32.0 ± 12.8*	419.4 ± 153.0*	219.9 ± 25.0*	536.5 ± 254.9*
Unstimulated cells (control)	2.7 ± 5.0	48.7 ± 12.7	3.4 ± 5.6	119.8 ± 28.1
IL-10 (pg/ml) ± SD				
Sa-α11				
PL	0.0	11.6 ± 10.9	0.0	1.3 ± 2.2
B	9.8 ± 7.0	82.5 ± 43.2*	0.0	196.9 ± 168.7
Sa-α21				
PL	34.9 ± 37.7	56.3 ± 19.6*	0.0	72.7 ± 13.4*
B	85.5 ± 40.6*	160.4 ± 46.2*	44.9 ± 35.7*	241.3 ± 79.1*
Unstimulated cells (control)	6.5 ± 9.2	4.4 ± 10.7	1.0 ± 1.8	18.8 ± 16.2

All data are the means ± SD for four measurements

* A significant differences (*p* < 0.05) in the concentration of cytokines compared to control

**Table 4:** The cytokines production by mouse PMN and Mo after their 4 h contact with live staphylococci growing as planktonic (PL) or biofilm (B) form

Type of culture	TNF-α (pg/ml) ± SD		IL-10 (pg/ml) ± SD	
	PMN	Mo	PMN	Mo
*S. aureus* 8325-4				
PL	3.2 ± 5.5*	0.0	32.1 ± 16.7*	0.0
B	0.0	0.0	30.9 ± 20.2*	0.0
*S. aureus* Wood 46				
PL	0.0	0.0	12.5 ± 14.7	32.1 ± 55.6
B	0.0	38.9 ± 40.8	16.8 ± 19.6	46.6 ± 40.4
Sa-α11				
PL	0.0	0.0	19.8 ± 15.3*	73.5 ± 108.1
B	0.0	0.0	78.0 ± 22.6*	73.6 ± 92.6*
Sa-α21				
PL	232.7 ± 50.5*	111.5 ± 17.0	558.1 ± 84.5*	628.1 ± 175.1*
B	178.0 ± 32.0*	58.8 ± 17.1	616.5 ± 135.8*	469.4 ± 28.9*
Unstimulated cells (control)	53.6 ± 32.1	77.2 ± 23.9	0.0	0.0

All data are the means ± SD for four measurements

* A significant differences (*p* ≤ 0.02) in the concentration of cytokines compared to control

Only the filtrates obtained from the cultures of clinical strains exhibited the ability to stimulate the leukocytes to the production of IL-6 and IL-10 (Table 3). Moreover, it should be noted that biofilm filtrates demonstrated stronger stimulatory activity than planktonic. To exclude the possible impact of *S. aureus* proteases (probably present in the bacterial filtrates) on the cytokines level, a sample of recombinant mouse IL-10 was preincubated with all four staphylococcal cultures’ filtrates. No influence on its activity measured in an ELISA assay was observed (data not shown).

The results of the production of the above cytokines by leukocytes stimulated with live bacteria are presented in Table 4. Interestingly, both forms of clinical strain Sa-α21 culture (biofilm, planktonic) demonstrated the best activation potential of the cells, which was revealed by the production of TNF-α as well as IL-10. The presence of IL-6 in the supernatants obtained from cells stimulated with bacteria was not detected, though the concentration of this cytokine reached 71.1 ± 31.3 and 58.0 ± 11.7 pg/ml, respectively, for control PMN and Mo.

## Discussion

The idea of the presented study originated from the belief that microbial biofilm is not a structure fully isolated from the external environment. Bacterial cells deposited on the surface and embedded/covered by slime are still able to release, spontaneously (during cell growth and division) or purposely, some components and metabolites. These products can affect the outside conditions, thus they may be very important for pathogens and their interaction with host cells (Jones et al. 2005; Kumar et al. 2004; Wang et al. 2000). One of the most important mechanisms of the anti-microbial defense is the activity of leukocytes, including the secretion of cytokines/chemokines (Crozat et al. 2009; Londono et al. 2008). In the present report, we decided to check these aspects of leukocytes’ activity, after their direct contact with bacterial biofilm or its metabolic (released) products. Unfortunately, the observed differences in cytokine/chemokine secretion in response to biofilm bacterial cultures/filtrates compared to planktonic did not represent one common profile, but depended on a tested cytokine type as well as given bacterial strain properties. Therefore, it is not possible to say which form of bacteria—planktonic or sessile is a better stimulus for the secretory activity of immunocompetent cells. This conclusion is not surprising, if we consider two models of infections caused by mucosal biofilms proposed by Dongari-Bagtzoglou (2008). Bacterial biofilm is able to either enhance or inhibit the host pro-inflammatory response, depending on many factors, e.g. environmental characteristics, stage of infection, bacterial profits. It may also reflect the characteristics of the bacterial strain, as was observed in this study.

Examined MIP-1α and MCP-1 belong to one of the four known groups of chemokines, called subfamily CC and are recognized by receptors called CCR, localized on such cells as monocytes, lymphocytes, natural killers and PMN. It makes them potent chemoattractants for many immunocompetent cells at the initial inflammatory response (McLoughlin et al. 2006, 2008). Therefore, as it was shown in this study, high, quite stable MIP-1α and MCP-1 production after cell induction, independently of the kind of stimuli (bacterial strain and type of culture/filtrate—biofilm or planktonic), seems advantageous for host defense.

Complex interactions between pro-inflammatory and anti-inflammatory mediators permit to keep a proper balance and do not allow the development of pathological processes destroying our tissues (Adib-Conquy et al. 2003; Hessle et al. 2005). Hessle et al. (2005) paid attention to the differences in the cytokines’ production between the cells induced by Gram-positive or Gram-negative bacteria. Gram-positive bacteria stimulated the secretion of more TNF-α, whereas Gram-negative microbes tended to stimulate IL-6, IL-8 and IL-10. In our study such cytokines as TNF-α, IL-6 and IL-10 were also estimated. Weak production of IL-6 and IL-10 (Table 3) seems to confirm the previous observations, but we also proved significant differences in the cytokines’ secretion depending on various bacterial strains within the same species. Generally, clinical strains and their filtrates appeared to be better stimuli for leukocytes to produce cytokines than reference strains. Probably, it was dependent on bacterial components released to the culture medium, which can play a role of modulins. It was confirmed that Gram-positive cell wall components, such as PG and LTA, were able to induce cytokines’ production, including TNF-α, IL-6 and IL-10 (Jones et al. 2005; Kumar et al. 2004; Morath et al. 2001; Wang et al. 2000). In our study, LTA release from clinical *S. aureus* strains was stronger even 9.5 times (for planktonic cultures) and 20 times (for biofilm cultures) in comparison to laboratory strains. Indeed, PG was secreted on a similar level for both types of strains, but the differences in LTA liberation were evident and could influence leukocytes’ stimulation. Moreover, Jones et al. (2005) suggested that short-chain-length form of staphylococcal LTA can play a role of the most important inflammatory mediator and act as a Gram-positive equivalent of lipopolysaccharide.

In conclusion, the presence of many staphylococcal components/metabolites in filtrates obtained from *S. aureus* biofilm culture indicates a partially open character of this structure. Despite the cover of bacterial microcolony by EPS, many *S. aureus* virulence factors (PG, LTA, Hla) leak into the external environment and may play a role of bacterial modulins affecting host cells during infection, as we observed in this study in vitro. Staphylococcal strain-dependent and, to a lower extent, culture form-dependent influence on cytokine/chemokine production by PMN and Mo was proved. *S. aureus* clinical strains and their filtrates appeared to be more potent stimulatory factors in comparison to laboratory strains. The lack of one common profile in the differences between the activatory effects of planktonic and biofilm *S. aureus* cultures/filtrates indicates, that the disturbances in cytokines’ production could not be the only reason for the so-called “frustrated phagocytosis”, taking place during the contact of leukocytes with bacterial biofilm. Further studies on the other aspects of host cells—microbial biofilm cross-talk are needed to understand the pathomechanisms of biofilm-associated infections and to develop new strategies for their treatment.
